# Successful Contextual Integration of Loose Mental Associations As Evidenced by Emotional Conflict-Processing

**DOI:** 10.1371/journal.pone.0091470

**Published:** 2014-03-11

**Authors:** Ulrike Zimmer, Karl Koschutnig, Franz Ebner, Anja Ischebeck

**Affiliations:** 1 Department of Psychology, University of Graz, Graz, Austria; 2 Department of Radiology, Medical University of Graz, Graz, Austria; The University of Queensland, Australia

## Abstract

Often we cannot resist emotional distraction, because emotions capture our attention. For example, in TV-commercials, tempting emotional voices add an emotional expression to a formerly neutral product. Here, we used a Stroop-like conflict paradigm as a tool to investigate whether emotional capture results in contextual integration of loose mental associations. Specifically, we tested whether the associatively connected meaning of an ignored auditory emotion with a non-emotional neutral visual target would yield a modulation of activation sensitive to emotional conflict in the brain. In an fMRI-study, nineteen participants detected the presence or absence of a little worm hidden in the picture of an apple, while ignoring a voice with an emotional sound of taste (delicious/disgusting). Our results indicate a modulation due to emotional conflict, pronounced most strongly when processing conflict in the context of disgust (conflict: disgust/no-worm vs. no conflict: disgust/worm). For conflict in the context of disgust, insula activity was increased, with activity correlating positively with reaction time in the conflict case. Conflict in the context of deliciousness resulted in increased amygdala activation, possibly due to the resulting “negative” emotion in incongruent versus congruent combinations. These results indicate that our associative stimulus-combinations showed a conflict-dependent modulation of activity in emotional brain areas. This shows that the emotional sounds were successfully contextually integrated with the loosely associated neutral pictures.

## Introduction

It is difficult to ignore emotional content even if it is irrelevant. This fact is exploited by many commercials that present a basically neutral picture of a product with tempting emotional voices or music in the background (e.g., gentle, soft music that implies the soft feeling of clothes if washed with a certain washing agent). The reason why we cannot resist emotional distraction is that emotions can capture our attention (e.g. [1], [2]). Emotion can spread to meaningless objects like geometrical shapes [2], thus it is not necessarily required that both the distractor as well as the target convey an emotion. However, if they do both convey an emotion, they can be contextually integrated as par example a fearful voice with a fearful face [3]. Further, in the case of successful contextual integration, emotionally conflicting stimuli are processed differently than emotionally matching stimuli (e.g. [3], [4]). The presence of emotional conflict in a distractor-target combination can therefore serve as a diagnostic whether a loose mental association between an emotional distractor and a neutral target is sufficient for contextual integration.

A first study on emotional capture in the case of meaningless objects presented a series of color circles intermingled with pictures of a man either confronting the participant with a hand gun (fear stimulus) or sitting relaxed in a chair (neutral control, [2]). Even when focusing on the color of the circles, the task-irrelevant fear stimulus activated the amygdala to the same extent as when focusing on the emotion of the picture [2]. In another exemplary study, participants had to discriminate the spatial alignment of two laterally presented white bars while ignoring a picture of a fearful face. Despite the non-emotional task-focus, the amygdala was still found activated, at least as long the neutral bar discrimination task was not getting too difficult [5]. Though emotional processing might be in part depending on available attentional resources, it is widely accepted that emotion can capture attention [1], [5]–[8]. Important for our present question is, first, that emotional capture seems to spread from the emotional stimulus to a contextually-unrelated stimulus (e.g. from a threatening irrelevant hand gun stimulus to task-relevant colored circles) and that the spreading is highly unspecific. Second, the amygdala was found activated even when fearful emotional stimuli were task-irrelevant, indicating emotional capture.

In contrast, contextual integration is more specifically involved in emotional capture. This means that it should matter, if an emotional stimulus is connected with an emotionally matching or conflicting stimulus-part. Typically, studies that used contextually integrated stimuli used two emotions that were integrated into one multifaceted object, such as an emotional voice with a facial expression, or a word (happy/fear) overlaid on a facial emotional expression (happy/fearful) (e.g. [4], [9]–[14]). Similar to the previously discussed studies, one stimulus part was task-relevant, whereas the other stimulus part had to be ignored. When the stimulus combinations were presented with conflicting versus matching emotional information, reaction times increased [3], [4], [9], [10], [12]. On the neural level, activation was found to increase for the conflicting case in the dorsal part of the anterior cingulate cortex (dACC) [3], [9], the amygdala and medial orbitofrontal cortex [4], [9]. Thus, compared to emotional capture using unrelated stimulus parts, the activity of areas of preferred emotional processing (e.g. the amygdala) is modulated by the congruency of emotional context.

We used a Stroop-like conflict paradigm as a tool to investigate whether stimulus-parts that are comparatively loosely mentally associated such as an auditory emotion and a neutral visual target are contextually integrated. If they are integrated then it should matter whether the visual target is conflicting or matching with the emotional sound, that is, we should see a modulation of activity in emotional areas due to congruency.

While previous studies have mainly used stimulus materials which carried the emotional property of fear or anger, we here used the emotion of disgust as an auditory distractor. We expected to find activity related to disgust in the insular cortex, an area that shows some specificity for disgust similar as the amygdala does for emotional stimulus properties of fear and anger (e.g. [15], [16]). The activation within the insula cortex by emotion was also shown to be not dependent on the sensory input modality. For example, emotional stimuli expressing disgust activated the right insula independently if conveyed auditorily, visually, gustatorily or olfactorily [17]. Thus, when using disgust as an auditory distractor, we can expect to find the insular cortex activated. In the case of contextual integration we should find a modulation by conflict, whereas we would expect steady activation without modulation in the case of no integration.

In the present study, we created a contextual association by combining one of two visual neutral objects (apple with or without worm) with either a congruent or incongruent emotional sound (disgust/delicious). Importantly, first, the target “worm/no-worm” was only present in the visual, but not auditory stimuli. Second, only the sounds carried emotion. Thus, any emotional combination of the sound with the visual target would require a contextual association. More specifically, in a pretest, all pictures were rated as non-emotional or neutral, whereas sounds were rated as negative (disgust) or positive (delicious). In the fMRI-task, participants' attention was directed on the detection of the worm on the apple (presence/absence), while the emotional voice had to be ignored. We hypothesized that, in the case of contextual-association, auditory associations (like someone eating a good or bad apple) would be mentally created and then interact with the actually presented visual picture although they were only associatively related. Thus, we expected to find conflict-related increases of reaction-times as well as increases of activity in brain areas specific to the emotion involved (i.e. disgust in anterior insula; cf. [16]; delicious in the cerebellum; cf. [16], [18]). In contrast, if emotional sounds do only capture attention without creating contextual associations, there should no modulation by conflict.

## Materials and Methods

### 2.1 Ethics statement

The study was approved by the ethics committee of the Medical University Graz/Austria (ethics approval number 23-501ex10/11). After receiving an explanation of the procedures, all participants gave written informed consent.

### 2.2 Participants

Thirty-seven healthy, right-handed participants participated in a pretest outside the scanner judging the emotional quality of our stimulus material (see below). Another twenty-four healthy, right-handed participants (ages 18–35 years; 12 female) took part in the fMRI-experiment. Five participants had to be excluded due to excessive head movement (>2 mm) or bad behavioral performance (less than 70% correct responses), leaving 19 participants (nine men) for final functional MRI and behavioral analyses.

### 2.3 Stimuli & fMRI-Paradigm

The aim of this fMRI study was to assess whether emotional capture can result in contextual associations. The target (i.e. worm/no-worm) is exclusively present in the visual stimulus, whereas the emotion of the auditory stimulus has to be contextually associated to the visual target. The present study tested if the emotional meaning of the sound would lead to a contextual association between the emotional sounds and the neutral visual targets. Using a Stroop-like conflict paradigm as a tool, activation in emotional brain areas should be modulated by the emotional conflict only in the case of successful contextual integration. The fMRI-participants had to detect the visual presence or absence of a little worm on an apple preceded by an emotional sound. Importantly, there was no direct connection between the visual task and the emotional sound, like a voice saying “worm” or “no-worm”. Instead, we intended to explore a more implicit connection between the auditive and the visual stimulus events. Our two emotional sounds (delicious/disgusting) suggested a good clean apple (humming sound (‘mmh’) or a bad wormy apple (vomiting sound). To enhance this indirect multisensory connection, we tried to choose realistic visual and auditory stimuli as described in detail below in the stimulus section.

To investigate the emotional quality of the stimulus materials for the fMRI-experiment, thirty-seven participants were asked in a pretest first to name the emotion elicited by the unisensory presented sounds and pictures including the possibility of “no emotion at all”. Apple images and sound stimuli had to be rated on a scale of one (strongly positive) over three (neutral) to five (strongly negative) according to their valence. Worm-pictures as well as no-worm pictures were judged by the pretest-participants as emotionally neutral pictures (more results below). The sounds and all apple pictures (with/without worm) were then used in the main fMRI-experiment. Participants of the pretest were excluded from fMRI-participation.

**Figure 1:**
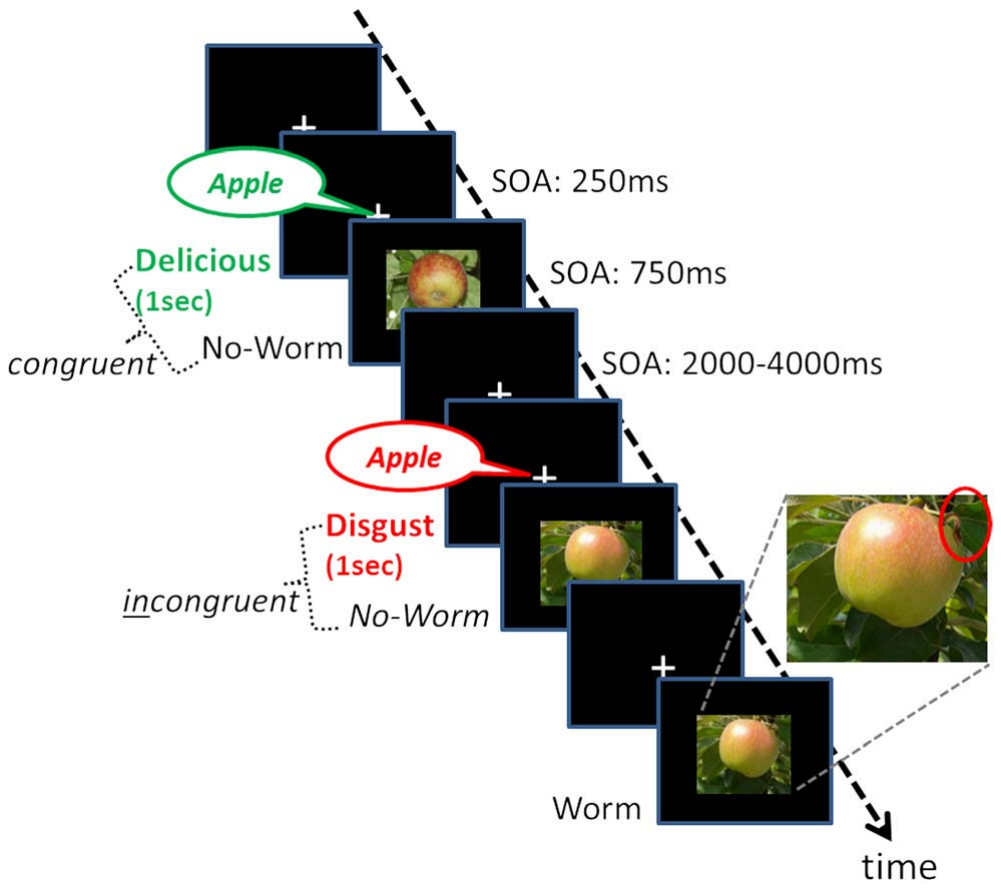
Task paradigm. An example of a stimulus sequence is shown. The task of the participants was to fixate on the central cross and detect the presence or absence of a little worm on an apple (50% probability each). Two-thirds of all trials were accompanied by an emotional sound (disgust/delicious) preceding the visual target stimulus by 250 ms. There were 50% incongruent and 50% congruent prosody-target combinations for each auditory emotional type. Participants were instructed to ignore the auditory sound and to focus on the search for the worm. (SOA =  Stimulus onset asynchrony).

Two photographs of a single apple hanging on a green tree branch served as apple pictures. They were presented in original view or as mirror images (see Figure 1), resulting in four slightly different apple pictures. When presented, the worm could appear on the apple at one out of four possible positions (upper left/right, lower left/right), always on the surface of the apple against a background of leaves. The total amount of presentations of each of these four apple and sixteen apple/worm combinations was counterbalanced in every run. Each picture was presented centrally for a duration of 750 ms, subtending a horizontal visual angle of 12° and a vertical visual angle of 12° with the apple in central position and covering 80% of the picture, thus corresponding to a realistically sized apple (width: ca.12 cm; height: ca.8.4 cm) when watched through the mirror on the head coil inside the scanner. The remaining 20% of the picture were covered by the leavy tree branch which held the apple. The worm size was adjusted after pilot experiments with the detection being relatively hard (length of worm: ca.3.4 cm; thickness: ca.0.83 cm). It appeared in one out of the four possible positions at a 4–5 cm distance from the geometric center of the apple. It should be noted that the pictures did not evoke emotions per se in the pretest.

The two different emotional sounds had a duration of 1 sec and preceded the apple-pictures in multisensory trials by 250 ms (Figure 1). They consisted each of two parts: an emotionally neutral female voice speaking the word “apple” overlaid by a sound of either vomiting or a deliciousness expressing crowing-humming sound (“mmh”). By overlaying the neutral “apple”-voice by both emotional sounds, we expected to increase the possibility for a contextual association for the emotional sounds and the neutral apple-pictures. Note that despite of this overlaid sound, the visual-auditory combinations need to be “contextually associated” as the task-relevant property the apple w/o worm is presented visually. The sound intensity for both of these combinations (disgust/delicious) was adjusted to 70 dB, consisting to 40% of the “apple”-voice and the emotional sound to 60% (percentages in units of overall loudness). To ensure that sounds were clearly audible during scanning, sound proofed fMRI-earphones were used which funneled sounds directly into each ear canal and additionally attenuated the surrounding scanner noise by a noise-reduction level of 29 dB (http://www.complyfoampro.com/products/canal-tips-original/). We did not further adjust the frequency-time structure, as its specific fingerprint determines the emotional character (delicious/disgust) of the resulting sound (cf. for happy/sad emotional sounds [19], [20], [21]). However, to exclude that the fMRI-results were due to differences in frequency-time structure, we compared only incongruent with congruent trials based on the same sound, thus subtracting away physical sound differences.

Each trial consisted of an apple-picture including either a little worm or not (“worm present”, “worm absent”). On two-thirds of the trials, the apple-picture was accompanied by one of the task-irrelevant emotional sounds (the onset preceding the picture by 250 ms, total duration 1000 ms) that conveyed disgusting or delicious emotional content. We kept the temporal onset between sound and picture constant to be able to subtract any possible preparation effects by comparing incongruent with congruent trials based on the same sound. The pairing of a “worm present”-picture with a disgusting sound as well as a “worm absent”-picture with a delicious sound were congruent multisensory combinations, as the information (good or bad apple) delivered by the sound was consistent with the picture. The other picture-sound combinations yielded the incongruent condition. In summary, the multisensory combinations consisted of equal parts of congruent and incongruent combinations, with half of each with a picture of “worm present” and “worm absent”. For the remaining one-third of all trials we included purely visual apple stimuli (without any preceding sound) to be able to subtract this condition from the corresponding multisensory responses, analogous to the approach used by Zimmer and colleagues [22], [23] with letter stimuli. Please note that such subtractions extract multisensory effects of the auditory emotion which had occurred either with or without the apple picture stimulus. The occurrence of all stimulus combinations (uni/multisensory and with/without worm) was randomized and unpredictable. The inter-stimulus-interval had a duration between 2000 ms to 4000 ms. Every subject completed eight runs of 60 stimuli, resulting in a duration of approximately 40 minutes (480 trials). Participants were instructed to ignore the voice and to press one of two buttons with their right index finger when they detected a worm on the apple, the other button when they decided there was no worm.

### 2.4 Image acquisition

Imaging was carried out in a 3 T Siemens Magnetom Tim Trio scanner (Siemens Medical Solutions, Erlangen, Germany) with a 32-channel head coil. Structural images for each participant were collected using an isotropic MPRAGE sequence with FOV 256 mm×256 mm×176 mm and a resolution of 1 mm×1 mm×1 mm. Functional BOLD (blood oxygenation level-dependent) contrast was obtained using a T2*-weighted EPI-sequence. The acquisition consisted of 34 transverse slices; thereby providing coverage of the whole cerebral cortex, acquired with a repetition time (TR) of 1.77 s and a TE of 25 ms. The in-plane resolution was 3 mm×3 mm, with a slice thickness of 3.75 mm.

### 2.5 Data analysis

#### 2.5.1 Pretest

Sounds and pictures were presented separately. After each stimulus presentation, each of the thirty-seven participants was first asked to name the emotion evoked by the presented stimulus including the possibility of “no emotion at all”. Then they rated the valence of the stimuli on a scale from 1 to 5 (1 = very positive; 2 =  positive; 3 = neutral; 4 = negative; 5 =  very negative). We counted how many of our participants named disgust-related or delicious-related emotions for the four presented stimulus types. Secondly, ratings of valence were each averaged over stimulus type (sound: delicious/disgust; picture: apple w/o worm) and across participants. T-tests were calculated for the estimated emotional valence of the sounds and pictures. Further, one-sample t-tests determined if the averaged estimated emotional valence values were significantly different from the numeric test-value “3” (equaling neutral perception).

#### 2.5.2 Behavioral data during scanning

Only trials for which the behavioral responses occurred between 200–1000 ms after target presentation were considered for further analysis (resulting in 99.33% included trials). Accuracy rates and reaction times (RTs) for the correct detection of the presence or absence of the worm on the apple were computed separately for the congruent, incongruent, and pure-visual trial conditions. To align the behavioral analysis as close as possible to the fMRI analysis, we subtracted the RTs and accuracy -rates of the visual-only apple stimuli (“worm present” and “worm absent” with no accompanying auditory component) from the responses of the corresponding multisensory stimuli. Analyses of variance (ANOVAs) and subsequent paired t-tests were then performed on these extracted RTs and accuracy-rates for the four multisensory conditions (delicious: no-worm/worm, disgust: no-worm/worm). These subtractions extract the multisensory effects of the auditory emotion which had occurred either with or without the apple picture stimulus.

#### 2.5.3 Functional data

The MRI data were analyzed using the software package SPM8 (http://www.fil.ion.ucl.ac.uk). The first four image volumes of both runs were discarded to allow for stabilization of longitudinal magnetization, leaving 598 volumes per run and participant. The remaining functional images were motion corrected to correct for head movement. The images were transformed (normalized) into MNI space [24], [25], using the mean of the functional volumes, and then smoothed with a Gaussian filter of 8 mm full-width at half maximum (FWHM) to increase the signal-to-noise ratio and to facilitate group analyses.

Statistical inferences were based on a random effects approach [26], which comprised three steps. First, for each subject, a design matrix was defined that modeled six event types which was derived from the crossing of the two factors of worm (present/absent) and stimulus type (visual-only/congruent/incongruent) using the canonical form of the HRF-response. In this first level design matrix, we included as a covariate of no interest the participant's response times in an event-related manner (i.e. for each trial the mean-averaged value of the participant's response-time for the respective condition was added). This covariate of no-interest should remove variance associated with response-related differences between the worm-absent and worm-present condition (search effect). To extract multisensory effects on auditory emotion in context of the visual apple/apple-worm pictures, we subtracted the activation in the pure visual condition from the activation in the respective multisensory condition (for example, the pure visual no-worm activation was subtracted from the no-worm/disgust regressor as well as from the no-worm/delicious regressor), resulting in four contrast images per subject (extracted contrasts). For the second-level group analyses, the four contrast-images of each of the 20 participants were used to create a flexible factorial model of SPM8 defined as interaction of the 2×2-factors target-presence (i.e. worm absent/present) and emotion (disgust/delicious) resulting in four regressors corresponding to each of the possible factor combinations.

#### 2.5.4 Definition of ROIs

The ROIs for testing emotional conflict were functionally defined. For disgust, we evaluated general effects of target presence vs. absence during visual search averaging over emotions (initial threshold: p<0.001 uncorrected reporting only clusters that surpassed a threshold of p<0.05, FWE-corrected at cluster-level). Significant cluster localized over emotional areas resulting from this contrast served as ROIs for further conflict specific testing. To determine ROIs for testing delicious conflict, the opposite functional contrast was carried out defining significant clusters (p<0.05; FWE-corrected) for worm-present compared to worm-absent trials while averaging over emotions. The resulting clusters over emotional brain areas were used for further analysis of delicious conflict.

#### 2.5.5 ROI –analyses

We tested for multisensory conflict effects in the suprathreshold clusters by comparing disgust/worm-present (congruent) versus disgust/worm-absent (incongruent) conditions in interaction with delicious/worm-present (incongruent) versus delicious/worm-absent (congruent) conditions. Note, that this is an orthogonal comparison as suprathreshold ROI-clusters were identified in a manner that is independent of emotion (a similar orthogonal analysis was used by Zimmer and colleagues [22]). The beta-values of each condition were extracted by using the complete cluster (predefined functional ROI) from each brain area for each condition (using the MarsBaR toolbox for SPM, http://marsbar.sourceforge.net/) and averaged. Accordingly, in this analysis we do not assess single voxel activations, but instead we want to know if the activation of the entire ROI is significant for interaction effects. Thus, no within-ROI multiple comparison corrections are necessary for this [27]. To gain further insight into the functional significance of the revealed brain activity in emotional conflict processing, we tested for correlations of the conflict-related brain activation (contrast values) across participants with the difference in reaction times between congruent and incongruent emotional stimulations.

#### 2.5.6 Auditory Cortex

The auditory cortex is an unisensory brain area which has been found to be involved in multisensory processing of neutral stimulus conflict [22], [23] as well as multisensory integration of unisensory stimuli [28]. It is therefore possible that emotional conflict evoked by emotional types of different sensory modalities (e.g. auditory/visual) may also activate unisensory areas. Thus, we also assessed the involvement of the auditory cortex in the present study. The ROI's of the left and right auditory cortices were anatomically and functionally defined by the overall effect of the task averaging over all regressors (p<0.05, FWE-corrected, at cluster-level).

## Results

### 3.1. Results of the pretest

The analysis of the emotional sound types indicated that the vomiting sound (overlaid with the neutral “apple” -voice) was named with disgust-related nouns (“disgust”, “aversion”) by 94.6% of the pretest participants (35 out of 37) and with fear by 5.4% (2 participants). In contrast, the humming sound overlaid by a neutral voice saying “apple”) was named by 83.7% participants (31 out of 37) with delicious-related words (“delicious”, “appetite”, “enjoyment of food”). The remaining 16.3% also rated the sound as positive, although not with specific delicious-related adjectives (“happy”, “joy”, “pleasure”). The valence of the stimuli were rated on a 1 to 5 scale (1 = very positive; 2 = positive; 3 = neutral; 4 = negative; 5 = very negative). The analysis for the vomiting sound revealed an average value of 4.2 (SD 0.56) whereas the humming sound was averaged to 1.97 (SD 0.60). Follow-up t-tests revealed that these ratings (for vomiting versus humming) were significantly different from each other (t(36)  = 13.71; p<0.001). Further, one-sample t-tests confirmed that the disgust as well as the delicious sound were significantly different from the neutral rating “3” (disgust sound: t(36) = 11.89, p<0.001; delicious sound: t(13) = 10.41, p<0.001).

In contrast to the sounds, participants complained about the impossibility to assign emotions to the apple-pictures (with and without worm), resulting in nearly only “no emotion” and “I really don't know”-answers. Correspondingly, the valence of both types of apple-pictures was estimated close to neutral (worm absence: mean = 2.75 (SD 0.83); worm presence: mean = 2.97 (SD 0.86)). Follow-up t-tests confirmed that these averages did not differ between each other (t(36) = 0.346; p = 0.955). Importantly, both averages did also not significantly deviate from the neutral rating “3” (absent worm: t(36) = 0.782, p = 0.183; present worm: t(36) = 0.190; p = 0.850). Thus, all apple stimuli (independent if presented with or without worm) are statistically perceived as equally neutral.

### 3.2. Behavioral Results during scanning

**Figure 2:**
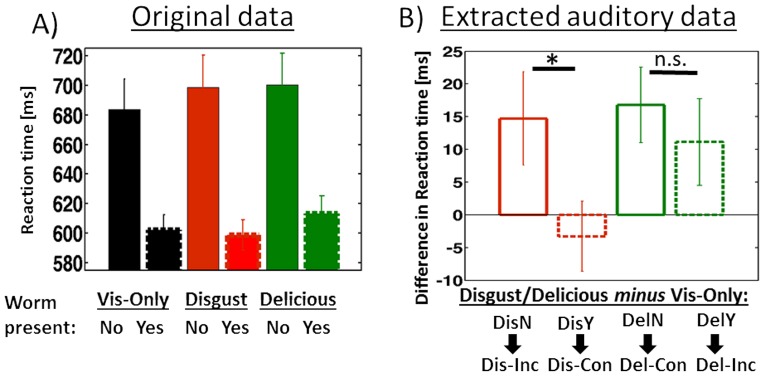
Behavioral results. A) Original data: Reaction times to the pure visual and multisensory stimuli for worm absent/present presentations indicate longer search times for worm absent compared to worm present trials. B) Subtracting pure visual stimulus conditions from multisensory reaction times in the worm-absent and worm-present trials. Abbreviations under the little arrows indicate whether the emotion/target-presentation results in a congruent or incongruent emotional stimulus combination. (Abbreviations: Dis = disgusting sound, Del =  delicious sound; N =  worm absent, Y =  worm present; Inc =  incongruent; Con  =  congruent).

Our fMRI-Participants were instructed to visually attend to the apple pictures and to detect the presence or absence of a little worm with a button press. The percentage of excluded trials due to RT's larger than 1000 ms was 0.67% over all participants. Search times were longer when the worm was absent compared to when it was present, which was reflected in a significant main effect of the factor worm absent/present (F(5,59) = 29.45; p<0.001, Figure 2A). Our main research interest was the multisensory influence of task-irrelevant emotional sounds on visual detection. We therefore extracted effects of auditory emotion from the visual apple/apple-worm pictures. That is, we subtracted the reaction time for the worm-present purely visual condition from the worm-present multisensory condition, and the reaction time for the worm-absent purely visual condition from the worm-absent multisensory condition. The resulting reaction times were entered into a repeated-measures ANOVA. A significant interaction of emotion by target absence/presence was observed (F(18) = 6.08; p = 0.038, Figure 2B). Subsequent paired t-tests for the disgusting emotional sound revealed that reaction times were significantly slower in the incongruent case (worm absent) (t(18) = 2.33; p = 0.032, Figure 2B). In contrast, there was no congruency effect for the delicious emotional sound (t(18) = 0.76; p = 0.454).

Accuracy rates were defined as behavioral responses for indicating correctly the absence of the worm in the no-worm condition or the presence of the worm in the worm-present condition. Accuracy -rates for each of the conditions were as follows: pure visual: Worm absent: 93.4% (SD: 16.6); Worm present: 94.2% (SD:7.7); disgust: Worm absent: 93,4% (SD:16.3); Worm present: 95.1% (SD: 4.6); delicious: Worm absent: 92.8% (SD: 16.1); Worm present: 95.3% (SD: 4.2). For follow-up statistics, accuracy-rates were analyzed by using a repeated-measurement ANOVA with the factors “Sound” (3 levels: silence, disgust, delicious) and “worm” (2 levels: absent, present). However, neither the interaction these factors nor the main effects revealed any significant effect. Additional analyses on the auditory extracted accuracy -rates were also not significant.

### 3.3. fMRI-results

The aim of the current study was to investigate whether a contextual association between an emotional distractor and a neutral target is sufficient to evoke emotional conflict. We expected emotional conflict to be present only in small focused emotional brain areas. Emotional brain areas were determined as the intersection of the brain areas activated during visual search (contrast worm-absent minus worm present) and the anatomical location of the respective area (e.g. insular cortex). Please note that areas indicative for visual search were identified averaged over emotions. In our ROI-analysis we then compared the influence of different emotions, so that this analysis is orthogonal to the contrasts that identified the areas activated during visual search. Here, we were first interested in the interaction between emotion and congruency, indicating emotional processing in the presence of conflict.

**Figure 3:**
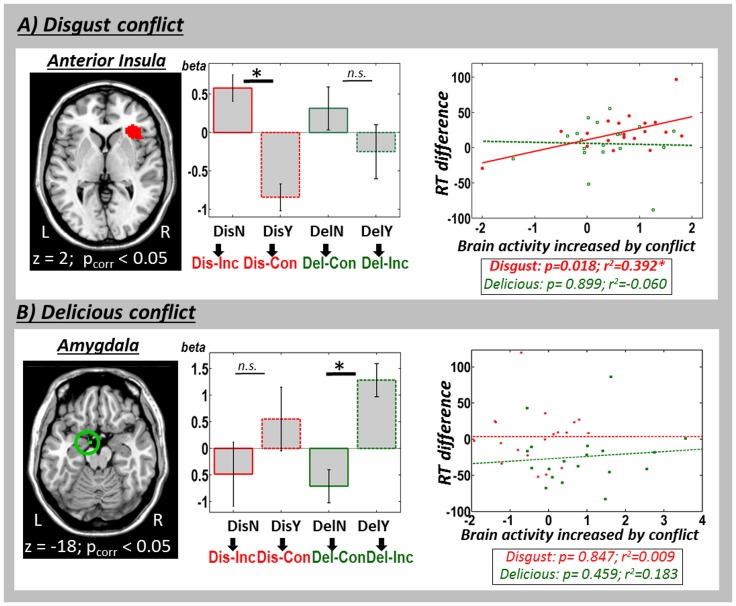
Modulation of brain activity in emotional areas by emotional conflict. A) The anterior insula showed a conflict-related modulation of activity only for disgusting sounds (left diagram). Behavioral differences in reaction time for incongruent versus congruent stimulation (y-axis) were positively correlated with increased conflict-activity (x-axis) when presenting disgust (right diagram; red dots and line), but not when presenting the delicious sounds (right diagram; green squares and line). B) In the amygdala, conflicting activity changes were found only for the delicious sound. Correlations of behavior and neural activity for emotional conflict were not significant. (Abbreviations as in Figure 2).

For the determination of emotional ROIs, we identified brain regions modulated by the presence or absence of the worm, averaging over emotional sounds and using the extracted auditory activity (Table 1). Importantly, this whole-brain voxel-wise comparison yielded enhanced activity for worm-absent versus worm-present trials in the insula cortex, an area known to be involved in disgust processing (e.g. [16]; see Table1A; cf. Fig. 3A). The activation within the insula cortex in the search task served as ROI for follow-up analyses as described in next section. Further, we found activation bilaterally in the visual cortex, in primary visual areas as well as in higher level visual regions as the parietal-occipital cortex (Table 1A). Thus, even after subtracting purely visually related activity, activity in the visual cortex was modulated by absent versus present extracted auditory activity, indicating a multisensory enhancement of visual search processes (see Table 1A).

**Table 1:** 

Absent vs. present target (unified over emotions)					
		Cluster		Peak-Voxel	
		p-value	k	t-value	x,y,z
Emotion specific area:	Anterior Insula	0.009	513	4.63	32,28,2
Further areas:	Anterior cingulate cortex	<0.0001	894	5.18	4,12,40
	Parietal occipital cortex	0.006	547	4.37	−2,−84,40
	Primary visual cortex	0.003	658	4.42	−6,−88,4

Present vs. absent target (unified over emotions)					
		Cluster		Peak-Voxel	
		p-value	k	t-value	x,y,z
Emotion specific area:	Amygdala	0.035	40	3.56	−8, −2,−18
Further areas:	none	---	---	---	---

Average overall extracted auditory conditions				
	Cluster		Peak-Voxel	
	p-value	k	t-value	x,y,z
Right Auditory Cortex	<0.0001	2291	11.60	54,−12,2
Left Auditory Cortex	<0.0001	1665	10.84	−56,−26,10

The aim of the current study was to investigate the possible contextual integration of auditory emotion with a neutral visual target. We expected this contextual integration to be indicated by a modulation of activity in emotional areas by emotional conflict (Insula cortex). We used the area of the Insula cortex which was activated by visual search (contrast worm-absent minus worm present) for subsequent ROI-analyses. In the ROI of the insula cortex, we were interested in the interaction between emotion and congruency. Beta-values extracted and averaged over the area of the entire ROI indicated an interaction of emotion by target-congruency. Specifically, activation in the anterior insula was increased for incongruent compared to congruent auditory disgust stimuli, but not for delicious stimuli. This result indicates that the insula is activated by stimulus conflict restricted to its specific emotion, namely, disgust (Table 2, Figure 3A central diagram). Control analyses revealed that all other brain regions which had remained in the visual search contrast (Table 1A) were not modulated by conflict-processing.

**Table 2:** ROI-Analyzes.

Interaction of emotion by target incongruency				
	*Disgust-Conflict*:		*Delicious-Conflict*:	
	t_(18)_-value	p-value	t_(18)_-value	p-value
**Anterior Insula**	**4.37**	**0.002**	*2.69*	*0.670*
**Amygdala**	1.643	0.118	**3.185**	**0.005**
Right Auditory Cortex	*1.019*	*0.427*	*0.565*	*0.628*
Left Auditory Cortex	*0.897*	*0.682*	*0.587*	*0.564*

Second, in the reverse contrast (worm present versus worm absent trials), an interaction between activation differences due to conflict and emotion was observed exclusively in the amygdala (Table 1B). Subsequent orthogonal t-tests indicated that the increase of activation in the amygdala was limited to the delicious emotion condition (incongruent (delicious/worm-present) versus congruent (delicious/worm-absent)) whereas the disgust emotion did not show a congruency effect (Table 2, Figure 3B).

We also investigated correlations of brain activity with behavior during emotional conflict processing. Increases of activity in the anterior insula were significantly related to the difference between congruent and incongruent disgust stimuli (worm absent/disgust) but not to reaction differences due to conflict in the delicious condition (worm present/delicious) (r^2^ = 0.392, p = 0.018, Figure 3A left diagram). In contrast, the activation within the amygdala did not show any significant correlation between reaction times and activation for any emotional type (Amygdala: disgust conflict: r^2^ = 0.009; p = 0.847; delicious conflict: r^2^ = 0.183; p = 0.549; Figure 3B left diagram).

**Figure 4:**
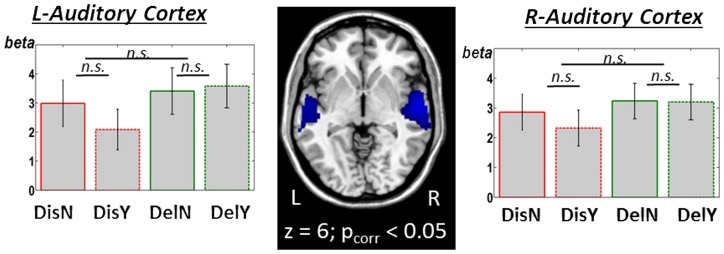
Auditory cortex activity elicited by the multisensory emotion conditions. There was no significant effect of incongruent vs. congruent stimulation in any emotion, nor a general effect of emotion itself. (Abbreviations as in Figure 2).

We also analyzed the involvement of unisensory auditory areas in the present study. Averaging over all stimulus types (emotions and target types) revealed an activation of the auditory cortex, bilaterally (Figure 4, Table 1C), with activity spreading from Heschl's Gyrus to large parts of the superior temporal gyrus. In these functionally defined areas, however, extracted auditory activity did not significantly vary for the different apple presentations (with/without worm) or for emotional type (Figure 4, Table 2).

## Discussion

We used a Stroop-like conflict paradigm as a tool to investigate whether an emotional auditory distractor and a neutral visual target could be contextually associated. The target “worm/no-worm” was only included in the visual stimulus-parts, but not in the auditory stimulus-parts. We hypothesized that, auditory associations (like someone eating a good or bad apple) would be mentally created and then interact with the actually presented visual picture (good/bad apple due to ab-/presence of the target-feature “worm”) although they were only associatively related. Importantly, in the case of integration, emotional conflict should matter.

We tested whether the associatively connected meaning of an ignored auditory emotion (delicious/disgust) with a non-emotional neutral visual target (i.e. the presence or absence of a little worm on an apple) would yield activations sensitive to emotional conflict in the brain. Pretests on the auditory and visual stimulus material evidenced that visual stimuli were perceived as neutral independent of the presence of the little worm. In contrast, disgusting sounds were rated similarly negative as delicious sounds were rated positive. There were two main results in the fMRI-experiment. First, there was stronger activity within the anterior insula when the disgust sound was in conflict with the visual target (disgust with worm absent) compared to the disgust sound matching the visual target (apple with worm present). Second, the amygdala showed increased activation when the delicious sound was presented with an incongruent target (apple with worm) compared to a congruent target (apple without worm). This conflict-dependent modulation of emotional activity indicates that the associatively related emotional sounds were successfully integrated with the neutral pictures.

### 4.1 Evidence for contextual integration

We argue that the modulation found in emotion sensitive brain areas due to congruency can be taken as evidence for contextual integration of emotion. First, previous studies indicated increased activity in emotional areas when producing emotional conflict in strongly contextually related stimuli, such as for example, stimuli consisting of an emotional facial expression and an emotional word [4]. Our results extend these findings by indicating that emotional contextual integration does also occur in loose mental associations. Second, we could only find a modulation of activity in emotional areas, but not in sensory areas (i.e. auditory cortex). It is possible that conflict activation in sensory brain areas are only observed with neutral stimulus combinations as the auditory cortex was found to be modulated in non-emotional multisensory integration of letter-pictures with conflicting or matching letter sounds [22], [29]. In the present study, however, there was a modulation exclusively in emotional, but not sensory brain areas. This could indicate that the associative contextual connection created here was based primarily on emotion.

In the present study, stimuli were not presented simultaneously. The emotional sound preceded the visual picture by 250 ms with a subsequent multisensory overlap of 750 ms. A recent study of neutral contextual integration showed that neutral letter-picture and letter-sound stimuli are best integrated when sounds are simultaneously presented with the picture [29]. In contrast, emotional integration seems to work best when sounds are preceding the visual stimulus. An EEG-study on emotional contextual priming using prosodic voice primes (anger, fear, happy) varied the duration of the stimuli (200 or 400 ms) which directly preceded a related or unrelated facial expression [12]. The authors observed a larger N400 for unrelated compared to related combinations only for prosodic primes with a duration of 400 ms. It is possible that it takes longer to recognize auditory stimuli when they carry emotional information. Thus, in the present study, the successful integration of the stimuli into an emotional context might have been promoted by the temporally leading and overlapping emotional sound.

### 4.2 Emotion specific effects

#### 4.2.1 Disgust

We observed differences in reaction times between incongruent versus congruent audiovisual pairs as well as an increase of activity in the anterior insula for the incongruent disgust (worm absent) combination compared to the congruent combination (worm present). Further, our correlation between reaction times and conflict activation within the anterior insula indicates that the activity in the anterior insula was linked to the behavioral performance on detecting conflicting disgust presentations. These results show that emotional context is processed in brain areas that show some specificity for the emotional content of the prosodic distractor. Importantly, our present results indicate that the auditory emotion is contextually integrated with the neutral picture also when distractor and target are only loosely mentally associated.

The anterior insula is one area out of a network of areas that show some specificity for disgust. It was found to be specifically involved when participants are exposed to disgusting odors or tastes compared to neutral stimuli [16], [30]–[33], disgusted facial expressions (fMRI [16], [34]–[38]; ERPSs: [39]) or disgusting pictures (e.g. maggots, rotten food, poor hygiene, [40]). Overlaying the results of 93 neuroimaging studies on emotional processing (including disgust) in four sensory modalities (vision, audition, olfaction, and gustation) identified the insula as a multisensory area for disgust processing and other negative emotions [17].

Importantly, we show for the first time that the insula activation is modulated when the disgusting stimulus is accompanied by an associatively related incongruent pictorial stimulus compared to a congruent one. While the involvement of the insula in presentations of disgusting emotions as well as other negative emotions is well established (e.g. [17], [41]), its role in emotional conflict processing has not yet been described. Most studies using Stroop-like tasks to investigate emotional conflict processing have used other emotions such as fear/angry/happy (audio-visual: e.g. [3]; visual: e.g. [4], [42]; auditory: e.g. [9]). Etkin and colleagues [4] observed that the subjective perception of fear/happy conflict measured by reaction time differences between incongruent and congruent stimulation correlated positively with the neuronal connectivity strength of the ACC with the amygdala, an area typical for the processing of fearful emotions. A recent fMRI-study [40], comparing disgust versus fear in brain activity indicated that the higher participants evaluated a picture as disgusting, the higher the insula region was activated. In our study, we found a positive correlation of insula activity with reaction times when visual targets were conflicting with task-irrelevant disgusting sounds. Therefore, although the disgusting sounds were the same, their processing in the insula was modulated by their congruency to the visual target.

#### 4.2.2 Delicious

We found an activity modulation in the amygdala by congruency for the delicious sound. This result seems surprising, as the amygdala is typically known as a detector of fearful emotion, or at least negatively valenced emotional stimuli (e.g. [6], [43]). However, some fMRI-studies indicated that amygdala activation might be independent of emotional valence [44], [45]. Recent neuroimaging studies on emotional conflict processing used integrated emotional stimuli (e.g., a facial expression overlaid by an emotional word or presented with a prosodic stimulation; e.g. [3], [42], [46]). Müller and colleagues [3] found that activity in the amygdala was stronger in incongruent than in congruent face/voice stimulations. However, less activation was observed when the face/voice combination consisted of only emotional content (e.g. facial fearful expression) combined with a neutral input (neutral voice). The authors concluded that amygdala activity was increased the higher the overall emotional content. Egner, Etkin and colleagues [4], [42] overlaid facial expressions with emotional visual words and found increased amygdala activation when the emotions of face and word were conflicting compared to a matching negative face/word stimulation. Thus, they extended the classical role of the amygdala from the processing of pure negative emotions to the processing of emotional conflict. Our present amygdala activation may therefore be explained as reflecting emotional conflict processing. Alternatively, some emotional studies have shown that negative emotions have a stronger impact on behavior as well as neuronal processing than positive emotions (e.g. visual detection of emotional faces [47]; visual distraction by emotional faces [48]). This phenomenon is in general explained by the evolutionary developed experience that a fast and immediate reaction on a negative stimulus (a snake, an angry opponent) can be life-saving and support the survival of the species. Thus, for the present study, it can be speculated that the disgusting sound might be per se a strongly negative emotion that already activates the amygdala, so that the congruency of the visual stimulus (apple w/o worm) becomes irrelevant (see also [3]). In contrast, when delicious sound is presented (a positive emotion) the congruency of the visual stimulation can still modulate amygdala activation, with increased amygdala activation for the incongruent compared to the congruent associative connection (see also [4]).

### 4.3. No specific involvement of unisensory areas in emotional conflict processing

In the present study, activity in unisensory brain areas was not modulated by emotional conflict implied via an associative connection. Activation in visual cortex was boosted during exhaustive search (worm-absent presentation versus worm-present) regardless of prosodic valence and emotional conflict. The activity in auditory cortex did not differ between the emotional types regardless if the worm was present or not. The finding fits very well with a previous fMRI study on emotional sounds [49] which revealed an area in auditory cortex (the emotional voice area EVA) specifically involved when processing emotional sounds compared to neutral sounds. Importantly, the authors [49] could not find any differential activity in EVA due the emotional valence carried by the sounds. It therefore seems plausible to assume that emotional conflict, as in the present study, also does not involve unisensory auditory areas. Furthermore, a recent study on emotional face/voice conflict [3] also did not find any specific modulation of unisensory areas during the processing of emotional conflict. It should be noted that this non-involvement of unisensory areas in emotional conflict stands in contrast to recent results that found increased activity in auditory and visual cortices with increasing conflict of emotionally neutral stimuli [22]. In summary, our study shows the multimodality and sensory independence of emotional conflict processing also for associative connections, extending previous findings on the processing of pure emotions [17].

## Conclusions

The present study investigated if an emotional distractor (disgusting/delicious sound) and a neutral target (worm absent/present on an apple) could be contextually associated (i.e. if the vomiting sound could be specifically connected with the presence of the worm, possibly by associating food poising due to a wormy apple). If this is the case, we expected a modulation of brain activity in emotional areas when the visual target is conflicting versus matching with the emotional sound. Results showed that during disgust, activity in the anterior insula was increased by conflicting compared to matching combinations. Further, insula activity during conflict was positively correlated with reaction times. Conflict of deliciousness led to increased activity in the amygdala. Our findings demonstrate that emotional conflict is effective, although the auditory and visual stimuli were only associatively connected. This underlines the efficacy of pairings between auditory emotion such as soothing and enjoyable music with neutral targets such as shopping goods that is exploited in commercials. Future studies could use and extend our results, by focusing exclusively on negative emotional sounds (disgust/anger/sadness) in combination with neutral visual target, to test for the specificity of the insula activity.
